# Establishing Sensory Neurons as Therapeutic Targets in Peripheral Neuropathy Driven by Polyglutamine Expanded Murine ATXN3

**DOI:** 10.1002/ana.78317

**Published:** 2026-07-31

**Authors:** Juan P. Mato, John M. Hayes, Jacen Emerson, Maeha Sridharan, Toluwani Adelaja, Arsal Naeem, Alexandra F. Putka, Hayley S. McLoughlin

**Affiliations:** ^1^ Department of Neurology University of Michigan Ann Arbor MI USA; ^2^ Neuroscience Graduate Program University of Michigan Ann Arbor MI USA; ^3^ Department of Human Genetics University of Michigan Ann Arbor MI USA

## Abstract

**Objective:**

Repeat expansion mutations underlie diverse neurogenetic disorders, many involving the peripheral nervous system. Despite peripheral neuropathy being the most common neurological disorder worldwide, its contribution to repeat expansion diseases remains poorly defined, and robust preclinical models are limited. We established a translational framework to study peripheral neuropathy in spinocerebellar ataxia type 3 (SCA3), a fatal multisystem disorder caused by a pathogenic CAG expansion in ATXN3.

**Methods:**

SCA3 Knock‐In *Atxn3* ^*Q300/Q6*^ (KI) and *Atxn3* ^*−/−*^ Knock‐Out (KO) mice underwent sensorimotor nerve conduction and behavioral testing. Peripheral tissues were evaluated for SCA3‐related pathology, and transcriptional changes in KI dorsal root ganglia (DRG) were assessed by bulk RNA sequencing. To test therapeutic relevance, we generated a conditional “OFF” SCA3 knock‐in (cKI) mouse and crossed it with *Avil‐Cre* to selectively silence mutant ATXN3 in sensory neurons. Peripheral phenotyping was repeated in *Avil‐Cre;* cKI mice.

**Results:**

KI mice developed progressive sensorimotor deficits, peripheral histopathology, and RNA splicing dysregulation that parallel clinical features of SCA3. In contrast, KO mice showed normal peripheral nerve function, implicating toxic gain‐of‐function from mutant ATXN3 as the causative driver. Sensory neuron–specific silencing of mutant ATXN3 significantly ameliorated peripheral nerve abnormalities and DRG splicing dysregulation.

**Interpretation:**

These findings define a pathogenic role for repeat expansion–driven peripheral nerve degeneration in SCA3, identify sensory neurons as key therapeutic targets, and provide a preclinical platform for developing peripheral interventions in SCA3 and related repeat expansion disorders. ANN NEUROL 2026;100:1039–1054

## Graphical abstract

Repeat expansion disorders frequently involve peripheral neuropathy, yet mechanisms remain unclear. Using a spinocerebellar ataxia type 3 (SCA3) Knock‐In *Atxn3* ^*Q300/Q6*^, we identify progressive sensorimotor deficits, peripheral nerve pathology, and dorsal root ganglia RNA splicing dysregulation. *Atxn3* ^*−/−*^ Knock‐Out mice show normal function, supporting a toxic gain‐of‐function mechanism. Sensory neuron‐specific silencing of mutant ATXN3 ameliorates neuropathy and molecular defects. These findings establish a robust model of SCA3 peripheral neuropathy and highlight sensory neurons as promising therapeutic targets for repeat expansion diseases.

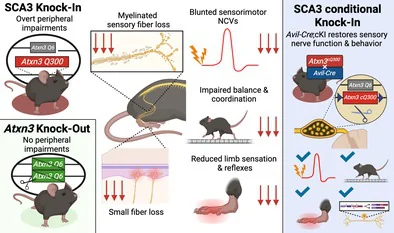

Repeat expansion mutations are major pathogenic drivers of several neurogenetic diseases with documented peripheral nerve involvement, including myotonic dystrophy, Friedreich's ataxia, and certain spinocerebellar ataxias.^1^, ^2^, ^3^, ^4^, ^5^, ^6^ Peripheral neuropathy is the most prevalent neurological disorder worldwide, presenting with sensory, motor, and autonomic deficits resulting from damage to the peripheral nervous system.^7^ Sporadic and inherited causes of peripheral neuropathy including diabetes, chemotherapy, transthyretin familial amyloid neuropathy (ATTR), Charcot–Marie–Tooth disease (CMT), and giant axonal neuropathy (GAN) have been well‐characterized. However, whether repeat expansion mutations directly cause peripheral neuropathy and, if so, how much neuropathy contributes to overall disease burden and progression remains a critical gap in the field.^8^, ^9^, ^10^, ^11^, ^12^

Mouse models provide a powerful platform to address this gap. They offer precise genetic control, uniform backgrounds, and controlled environments, enabling longitudinal assessments and direct tissue access. Such advantages permit mechanistic and therapeutic studies that are challenging in heterogeneous patient cohorts. However, few animal studies have comprehensively assessed neuropathic features in repeat expansion disorders, limiting mechanistic insights and therapeutic discovery.

As a framework to study repeat expansion‐associated peripheral neuropathy, we focused on the most common dominantly inherited ataxia, spinocerebellar ataxia type 3 (SCA3).^13^ This universally fatal neurodegenerative disease is caused by a CAG repeat expansion in exon 10 of the ubiquitously expressed *ATXN3* gene, producing a polyglutamine (polyQ)‐expanded ATXN3 protein.^13^ Patients develop progressive cerebellum, brainstem, and spinal cord atrophy alongside ataxia that impairs muscle control, coordination, and ultimately respiratory function.^13^, ^14^ Although clinical studies report peripheral nerve abnormalities in SCA3, findings vary, from mild sensory disturbances to pronounced nerve conduction deficits.^3^, ^15^, ^16^, ^17^, ^18^, ^19^, ^20^, ^21^, ^22^, ^23^ Importantly, reduced sciatic nerve volume has been observed in pre‐ataxic individuals, suggesting peripheral pathology can precede motor symptoms.^17^ In the central nervous system (CNS), accumulation of polyQ‐expanded ATXN3 drives toxic gain‐of‐function mechanisms including transcriptional dysregulation and protein aggregation.^13^, ^22^ Human postmortem peripheral tissues, particularly dorsal root ganglia (DRG) sensory neurons, exhibit similar ATXN3 accumulation alongside neuronal loss and structural nerve damage.^18^, ^24^ These findings implicate sensory neurons as contributors to SCA3 pathophysiology, yet peripheral involvement has remained underexplored in clinical and preclinical research.

Several SCA3 mouse models have been well characterized with respect to motor impairments and CNS pathology; however, they lack longitudinal, quantitative assessments of neuropathic phenotypes.^14^, ^25^, ^26^, ^27^ To establish the first robust animal model of SCA3 peripheral neuropathy, we systematically characterized neuropathic phenotypes in a SCA3 Knock‐In mouse that endogenously expresses one normal *Atxn3* CAG_6_ allele alongside a mutant CAG_300_ allele, a repeat hyperexpansion necessary to drive robust, progressive phenotypes over an accelerated disease timeline.^26^ These mice developed progressive sensorimotor deficits that mirror the human condition. Parallel studies in *Atxn3* Knock‐Out mice demonstrated that ATXN3 loss‐of‐function is well‐tolerated. We further developed a novel conditional “OFF” Knock‐In mouse, enabling sensory neuron‐specific silencing of mutant ATXN3 via Cre recombination. Silencing the polyQ‐expanded protein in sensory neurons mitigated sensory nerve conduction abnormalities and sensorimotor behavior deficits. These results highlight the contribution of peripheral neuropathy to a repeat expansion disorder and underscore the therapeutic potential of sensory neuron‐targeted interventions for SCA3 and related repeat expansion disorders.

## Methods

### Mice

Animal procedures adhered to the United States Public Health Service's Policy on Humane Care and Use of Laboratory Animals and approved by the University of Michigan Institutional Animal Care and Use Committee. Mice were housed with a 12‐h light/dark cycle and access to food and water ad libitum. Both sexes were included. The Knock‐In *Atxn3* ^*Q300*^ (KI) mice carry a hyperexpanded CAG_300_ tract in one endogenous *Atxn3* allele alongside a CAG_6_ allele. *Atxn3* Knock‐Out (KO) mice have a homozygous deletion of *Atxn3*. Genotyping was done as previously described.^26^ To investigate mechanistic questions in SCA3, we generated a conditional Knock‐In *Atxn3* ^*cQ300/Q6*^ (cKI) in which LoxP sites flank exon 4 of the mutant *Atxn3* ^*Q300*^ allele. Cre‐mediated excision of exon 4 causes a change in amino acid sequence at residue 78 and early truncation of the ATXN3 10 amino acids later. cKI mice were genotyped identically to KI mice. To selectively silence *Atxn3* ^*Q300*^ in sensory neurons, cKI mice were crossed with an *Avil‐Cre* mouse (JAX stock #032536). *Avil‐Cre*; cKI mice were crossed to tdTomato reporter mice (JAX #007914), allowing visualization upon Cre recombination. TdTomato genotyping was performed as previously described.^28^ All strains were maintained on a C57BL/6N background.

### Immunohistochemistry

Frozen sciatic nerves, DRG, and spinal cord sections from 40‐week KI and age‐matched wild‐type (WT) littermates underwent antigen retrieval in 80°C sodium citrate (30 min) and incubations in 1% H_2_O_2_ (30 min) and 5% goat serum in Tris B (1 h) before incubation in anti‐ATXN3 primary antibody (1H9, 1:500, MAB5360, Millipore; overnight). After biotin anti‐mouse secondary antibody (1:1000, 30,044, Vector Labs; 1 h) and ABC solution incubation (PK‐4000, Vector Labs; 1 h), slides underwent DAB reaction (3,3′‐diaminobenzidin; SK‐4100, Vector Labs), dehydration, and coverslipping (DPX, 13510, Electron Microscopy Sciences). Images were taken on a Nikon Ti2 Widefield microscope.

### Nerve Conduction Studies

Nerve conduction studies assessed sensorimotor nerve function. Mice were anesthetized with isoflurane (VetOne) and placed under a heating lamp to maintain body temperature. Recordings were collected using the UltraPro S100 EMG/NCS (Natus Medical). Sural nerve SNCVs and SNAPs were measured by applying antidromic supramaximal stimulation at the ankle with stainless steel needle electrodes (Natus Medical) and recording from the dorsum of the foot. SNCVs were determined by dividing the length (mm) between the recording and stimulating electrodes by the sensory nerve action potential take‐off latency (m/s) to reach the recording electrode. MNCVs and CMAPs were recorded from the sciatic nerve by applying orthodromic supramaximal stimulation to both the sciatic notch and ankle and recording from the dorsum of the foot. The distance between the sciatic notch and ankle electrodes (mm) was divided by the differences in the take‐off latencies (m/s) to calculate MNCV. Experimenter was blinded to genotype during procedures.

### Behavioral Assessments

Longitudinal behavior testing was performed at 16, 24, and 40 weeks of age in KI mice and 8, 16, and 24 weeks in KO mice. Weights were recorded at each timepoint. Balance beam testing involved two consecutive days of familiarizing with crossing a 5 mm balance beam. On the third day, latency to cross the beam was recorded in two trials and averaged. Falls were scored as 30 s. Accelerated Rotarod testing subjected mice to a 4–40 rpm accelerating rotarod (Med Associates, Inc.) for three, 5‐min trials/day over 4 consecutive days. Latency to fall was recorded from each trial, with five minutes between trials. Latencies from each day were averaged. Von Frey tests involved three consecutive days of 45‐min habituations in wire‐mesh bottom plexiglass cages (Ugo Basile, VA, Italy). Following habituation on the third day, a calibrated logarithmic series of monofilaments (Ugo Basile, VA, Italy), each with a set force (g) applied upon buckling, was applied to the plantar surface of mouse hindpaws. A rapid withdrawal or grooming of the hindpaw was marked as a positive response. The 50% g threshold was determined for each tested timepoint. Following identical habituation procedures to the Von Frey protocol, thermal hindpaw withdrawal latency tests used a 60% infrared light source to deliver heat to the plantar surface of the hindpaw. Seconds between heat activation and paw withdrawal was recorded with an analgesia meter (Ugo Basile, VA, Italy). A total of 5–6 responses from alternating paws were averaged.

### Sensory Fiber Density Measurements

Sural nerves were dissected and postfixed in 2.5% glutaraldehyde and 4% paraformaldehyde. TEM processing was done by the University of Michigan's Microscopy Core according to their protocol. Myelinated axon densities were measured on 40× images by fiber counting and area division (ImageJ). Diameters of 300 randomly selected axons per mouse were measured in ultrathin sections (JEOL‐JEM‐1400, MyelTracer).^29^ Experimenters were blinded to genotype.

Intraepidermal fiber densities were completed by postfixing plantar footpads in Zamboni fixative for 1 hour immediately after dissection (NC9335034, Newcomer Supply) and cryopreserving overnight. Fixed footpads were cryosectioned into frozen 30 μm sections before they were blocked in Triton X‐100 and 5% BSA and incubated overnight in anti‐ PGP9.5 primary antibody (1:2000, 50–553‐564, Proteintech). Sections were then incubated in Alexa Fluor secondary antibody (1 h, 1:2000, Sigma, D9564) nuclei were stained with DAPI before mounting with Prolong Gold Antifade Reagent (P36930, ThermoFisher). Intraepidermal fiber densities were quantified per European Federation guidelines and averaged across sections; experimenters were blinded.^30^

### RNA Extraction and Bulk RNA Sequencing

Flash‐frozen DRG from 40‐week KI and WT and 36‐week *Avil‐Cre*‐;WT, *Avil‐Cre‐*;cKI, and *Avil‐Cre+*;cKI were pulverized in liquid nitrogen before and homogenized in TRIzol (Invitrogen, 15596018). RNA was extracted (RNeasy Plus Mini Kit, Qiagen, 74134) per the manufacturer's protocol. Samples were submitted to the University of Michigan Advanced Genomics Core for library preparation and Illumina Next Generation Sequencing on a NovaSeq Flowcell. Libraries were prepared using NEBNext Poly(A) mRNA Magnetic Isolation Module (NEB, E7490) and NEBNext UltraExpress RNA Library Prep Kit (NEB, E3330). FASTQ files provided by the University of Michigan Advanced Genomics Core underwent quality control using FastQC (version 0.12.1, RRID: SCR_014583) to ensure all samples used had sufficient read depth (>75 million paired‐end reads) and check the adapter content. Trimming was performed using fastp (version 1.1.0) to reduce adapter content below 20%. All FASTQ files had <20% adapter content, so no trimming was performed. FASTQ files were aligned to the GRCm39 mouse reference genome using STAR (version 2.7.10b, RRID: SCR_004463). Differential gene expression was performed in RStudio (2025.05.0 + 513, RRID: SCR_000432; R version 4.4.2, RRID: SCR_001905) using DESeq2 (version 1.46.0, RRID: SCR_015687). Genes were considered differentially expressed if they passed a significance threshold of Padj <0.05 and log2FC > |1.5|. g: Profiler [(Kolberg et al., 2023), RRID: SCR_006809] was used for all gene ontology (GO) analyses. Alternative splicing analysis was performed using rMATS (version 4.1.2, RRID: SCR_023485). Splicing events were considered significant if they passed a significance threshold of FDR <0.05 and ΔPSI > |0.1|. All ΔPSI values are converted from a ratio to a percentage with a threshold‐adjusted ΔPSI > |10%| accordingly.

### Immunofluorescence

Brain, spinal cord, sciatic nerve, and DRG sections from 4‐week *Avil‐Cre*; *TdTomato*; cKI mice were blocked (1 hour, M.O.M kit, Vector, FMK‐2201). Brain sections were treated with antibodies for NeuN (1:200, Abcam, ABN78A4) and TdTomato (1:1000, Fisher, 50‐167‐1121). Sciatic nerve sections were treated with antibodies for S100β (Schwann cells, 1:200, Abcam, ab52642) and TdTomato. DRG sections were treated with antibodies for NeuN, S100β, and TdTomato. Spinal cord sections were treated with antibodies for NeuN, TdTomato, and CGRP (sensory axons, 1:500, Invitrogen, PA5‐114929). Alexa Fluor secondary antibodies (1 h, 1:2000, Sigma, D9564) were applied followed by DAPI (15 min). Slides were coverslipped with ProLong Gold and imaged as Z‐stacks on a Leica Stellaris‐5 Confocal microscope.

### Western Blotting

DRG lysates were homogenized in RIPA + PI buffer and protein concentrations were quantified per manufacturer's instructions (Pierce BCA Kit, Thermo Scientific, 23,227). Lysates were run on 4–20% SDS‐PAGE gels, transferred to 0.45‐μm nitrocellulose membranes, and imaged for total protein load (Direct Blue). Membranes were blocked (1 h, 5% milk in TBS‐T), incubated overnight in primary antibodies for ATXN3 (1H9, 1:1000, MAB5360, Millipore), GAPDH (1:10000, MAB374, Millipore), and 1C2 (1:1000, MAB1574, Millipore). Peroxidase‐conjugated anti‐mouse secondary antibody (1:5000; Jackson ImmunoResearch Laboratories) was applied, and bands visualized (GeneSys, SuperSignal West Femto, Thermo Scientific, 34,094). Band intensities were quantified (ImageJ), normalized to GAPDH.

### Statistics

Statistics were performed in GraphPad Prism 10.4.1. Normality was confirmed (Shapiro–Wilk), and outliers excluded via ROUT. Two‐way ANOVA with Bonferroni post hoc tests were used for timepoint comparisons; unpaired two‐tailed Student's t‐tests for single timepoint comparisons. Methods and sample size (n) are in figure legends. Data arereported as mean ± SEM; statistical significance notation in legends.

## Results

### SCA3 KI Mice Exhibit Sensorimotor Nerve Conduction Abnormalities Due to Mutant ATXN3 Toxic Gain‐of‐Function

Most SCA3 patients exhibit marked peripheral sensorimotor nerve conduction deficits, quantified by diminished sensory nerve action potentials (SNAPs) and compound muscle action potentials (CMAPs) that reflect underlying axon dysfunction.^3^, ^15^, ^16^, ^17^, ^18^, ^19^, ^20^, ^21^, ^23^, ^31^ In contrast, reductions in sensory and motor nerve conduction velocity (SNCVs and MNCVs, respectively) are milder, implying relative preservation of myelination. To model this, we utilized SCA3 Knock‐In *Atxn3* ^Q300/Q6^ (KI) mice, bearing an hyperexpanded CAG_300_ repeat mutant *Atxn3* allele. These KI mice exhibit progressive motor impairments and early mortality, phenocopying patient progression.^26^ Longitudinal nerve conduction studies at 16, 24, and 40 weeks tracked sensorimotor deficits through disease stages, alongside *Atxn3* KO mice to distinguish toxic gain‐of‐function from loss‐of‐function effects.^32^ SNAPs and SNCVs recorded from KI sural nerves were significantly blunted compared to WT controls, while KO mice showed normal function, indicating mutant ATXN3 induces progressive sensory axon loss/dysfunction through toxic gain‐of‐function. **(**Fig 1A–C). SNCV reductions in KI mice were mild compared to mouse models of demyelinating neuropathies, suggesting that demyelination is not the predominant pathology.^11^, ^33^ Motor nerve function, assessed by sciatic nerve CMAPs and MNCVs, revealed substantial reductions in KI, but not KO mice, implying motor axon loss/demyelination is also mutant ATXN3 gain‐of‐function‐driven **(**Fig 1D–F). Collectively, these findings confirm nerve conduction abnormalities in KI mice closely parallel SCA3 patient phenotypes and are driven by toxic gain‐of, not loss‐of, function.

**FIGURE 1:**
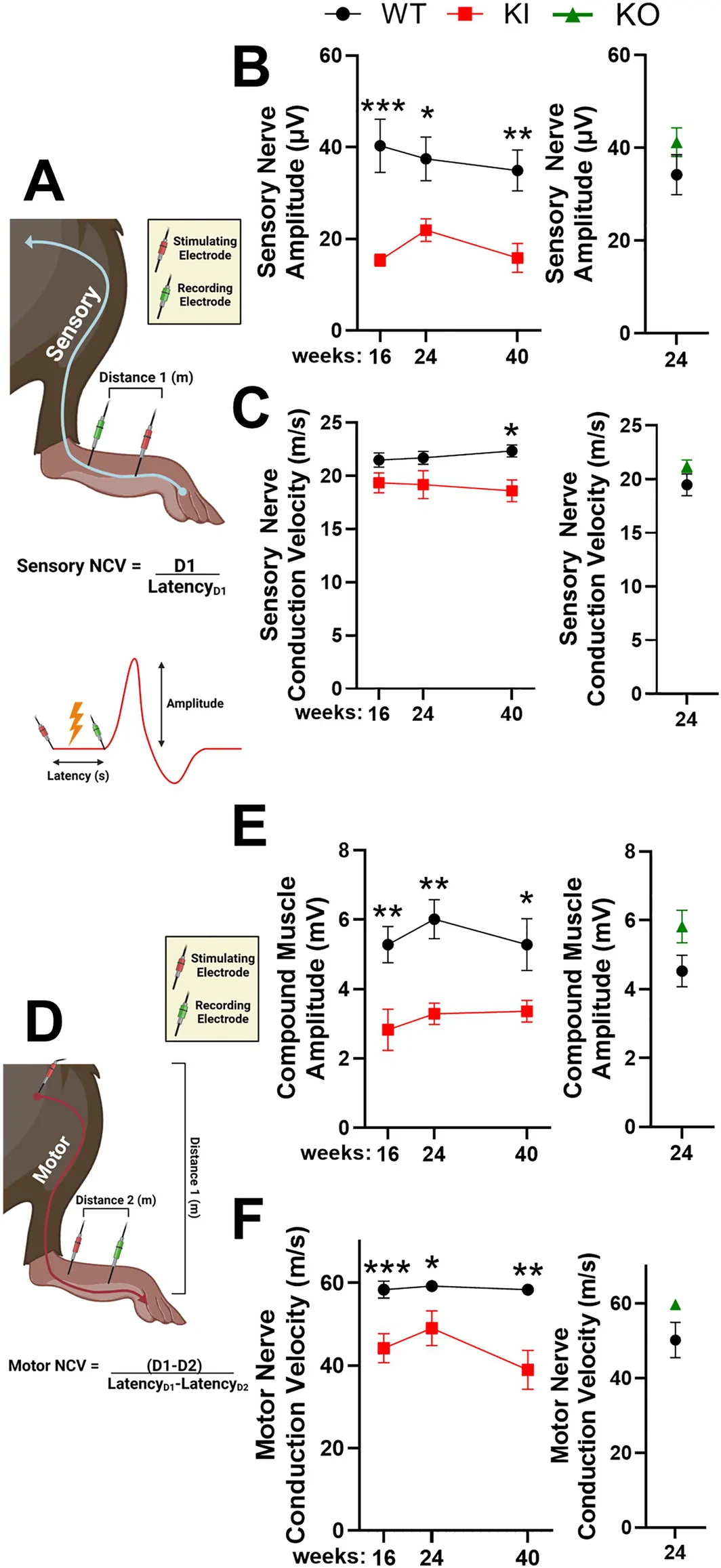
SCA3 KI mice exhibit sensorimotor nerve conduction abnormalities due to mutant ATXN3 toxic gain‐of‐function. (A) Schematic of antidromic supramaximal stimulation for recording sensory nerve conduction amplitude and velocity in mouse sural nerves. (B) Sensory nerve amplitudes recorded from 16‐, 24‐, and, 40‐week KI and 24‐week KO sural nerves in comparison to WT littermates (KI n = 4–9/genotype/timepoint; KO *n* = 6–9/genotype). (C) Sensory nerve conduction velocities recorded from 16‐, 24‐, and 40‐week KI, and 24‐week KO sural nerves in comparison to WT littermates (KI *n* = 4–9/genotype/timepoint; KO *n* = 6–9/genotype). (D) Schematic of orthodromic supramaximal stimulation for recording compound muscle action potentials and motor nerve conduction velocities in mouse sciatic nerves. (E) Compound muscle action potential amplitudes recorded from 16‐, 24‐, and 40‐week KI, and 24‐week KO sciatic nerves in comparison to WT littermates (KI *n* = 4–9/genotype/timepoint; KO *n* = 6–9/genotype). (F) Motor nerve conduction velocities recorded from 16‐, 24‐, and 40‐week KI, and 24‐week KO sciatic nerves in comparison to WT littermates (KI *n* = 4–9/genotype/timepoint; KO n = 6–9/genotype). All data are represented as mean ± SEM. KI data were analyzed via two‐way ANOVA with Bonferroni comparisons between genotypes (*p < 0.05, **p < 0.01, ***p < 0.001) and timepoints. KO data were analyzed with Student's unpaired t‐tests. [Color figure can be viewed at www.annalsofneurology.org]

### Mutant ATXN3 Toxic Gain‐of‐Function Induces Progressive Sensorimotor Deficits, Thermal Hypoalgesia, and Mechanical Desensitization in KI Mice

To determine whether nerve conduction abnormalities manifest relative disease features, we tested balance and coordination in KI and KO mice using balance beam and rotarod tasks. KI mice exhibited significant, progressive impairments starting at 24 weeks, while KO mice performed normally **(**Fig 2A,B **)**. Moreover, KI, but not KO mice, demonstrated reduced locomotion and rearing during the open field task **(**Fig S1A,B **)**. These KI impairments notably mirror temporal ataxic progression observed in SCA3 patients and recapitulate previous motor characterizations in this model.^26^ KI mice showed no overt anxiety‐like behavior in the open field task and performed identically to WT controls on the hindlimb clasping task, similarly to KO mice (Fig S1C,D). KI mice weighed less than WT controls beginning at 24 weeks, echoing SCA3‐related body changes (Fig S1E).^34^

**FIGURE 2:**
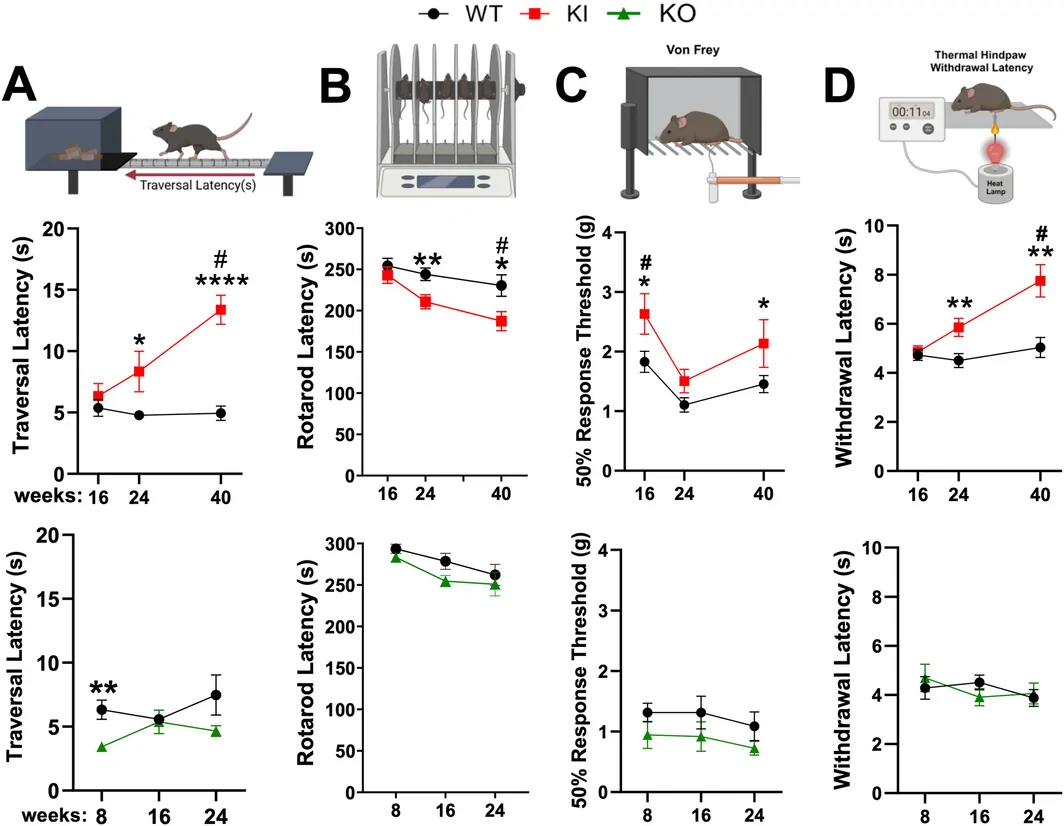
Mutant ATXN3 toxic gain‐of‐function drives progressive sensorimotor behavior deficits in SCA3 KI mice. (A) Latency to traverse balance beam assessed between 16‐, 24‐, and 40‐week KI and 8‐, 16‐, and 24‐week KO mice in comparison to WT littermates (KI *n* = 16–21/genotype/timepoint; KO *n* = 6–10/genotype/timepoint). (B) Latency to fall off accelerating rotarod assessed between 16‐, 24‐, and 40‐week KI and 8‐, 16‐, and 24‐week KO mice in comparison to WT littermates (KI *n* = 16–21/genotype/timepoint; KO *n* = 6–10/genotype/timepoint). (C) Von Frey 50% response thresholds assessed between 16‐, 24‐, and 40‐week KI, and 8‐, 16‐, and 24‐week KO mice in comparison to WT littermates (KI *n* = 6–12/genotype/timepoint; KO n = 6–8/genotype/timepoint). (D) Thermal hindpaw withdrawal latencies assessed between 16‐, 24‐, and 40‐week KI and 8‐, 16‐, and 24‐week KO mice in comparison to WT littermates (KI *n* = 6–12/genotype/timepoint; KO n = 6–8/genotype/timepoint). All data are represented as mean ± SEM and were analyzed via two‐way ANOVA with Bonferroni comparisons between genotypes (*p < 0.05, **p < 0.01, ****p < 0.0001) and timepoints (#*p* < 0.05). [Color figure can be viewed at www.annalsofneurology.org]

Sensory deficits in SCA3 patients often include diminished reflexes and limb numbness, contributing to mobility issues.^3^, ^21^, ^22^, ^35^ The SNAP deficits in KI mice (Fig 1B) and patients alike can be associated with symptoms such as tingling, areflexia, and numbness.^12^, ^22^ Von Frey tests showed KI mice had elevated mechanical response thresholds at 16 and 40 weeks, phenocopying patient mechanical insensitivity (Fig 2C). Thermal hindpaw withdrawal latency (THWL) assays showed delayed withdrawal responses between 24 and 40 weeks in KI mice, suggesting impaired nociception/reflexes (Fig 2D). KO and WT mice showed normal sensation, excluding sense loss as an ATXN3 knockout effect.

### SCA3 KI Mice Exhibit Pathological ATXN3 Accumulation in Peripheral Tissues alongside Loss of Myelinated Sensory Fibers and Intraepidermal Nociceptors

The striking peripheral dysfunction observed in KI mice prompted us to assess localization and accumulation of ATXN3 in KI sciatic nerves, DRG, and spinal cords at 40 weeks old (Fig 3A), a time coinciding with severe sensorimotor impairments. KI sciatic nerves exhibited marked ATXN3 accumulation within axons and Schwann cell regions (Fig 3B). DRG accumulations appeared in morphologically identified sensory neurons and satellite glial cells (Fig 3C), while dense ATXN3 puncta were detected in the spinal cord. (Fig 3D). These findings are consistent with corresponding tissues in SCA3 patients as well as neuronal and glial ATXN3 accumulation in disease‐vulnerable brain regions.^17^, ^36^, ^37^

**FIGURE 3:**
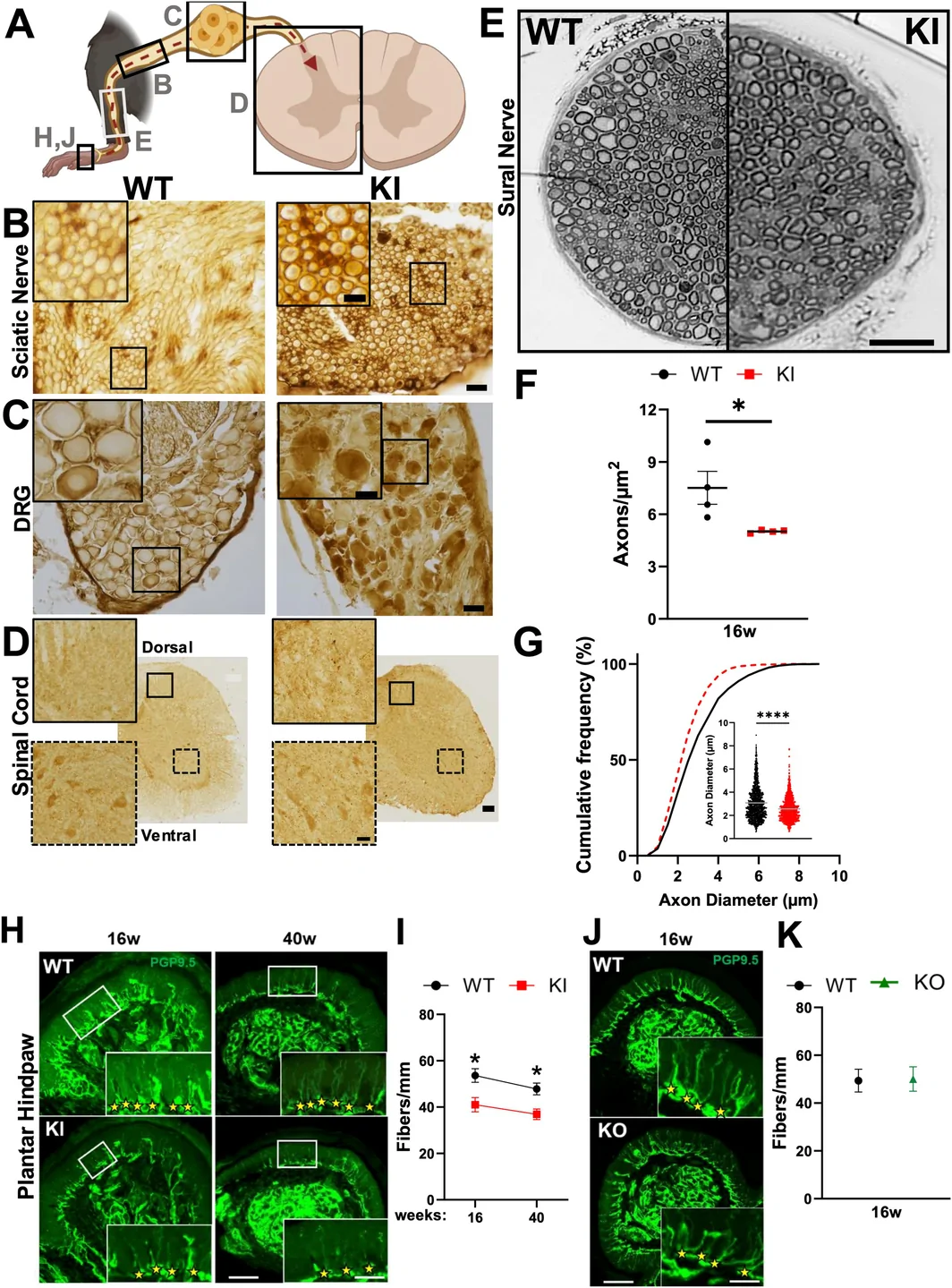
Peripheral nervous tissue shows pathological ATXN3 accumulation and sensory fiber loss in SCA3 KI mice. (A) Schematic of peripheral tissue regions immunohistochemically assessed for ATXN3 (anti‐1H9) expression. Boxes outline corresponding tissue types displayed in subsequent figure panels. (B) Representative images of anti‐ATXN3 immunohistochemistry from 40‐week WT and KI sciatic nerves (scalebar = 10 μm, inset scalebar = 2 μm). (C) Representative images of anti‐ATXN3 immunohistochemistry from 40‐week WT and KI lumbar dorsal root ganglia (scalebar = 100 μm, inset scalebar = 20 μm). (D) Representative images of anti‐ATXN3 immunohistochemistry from 40‐week WT and KI lumbar spinal cord (scalebar = 100 μm, inset scalebar = 30 μm). (E) Representative images of 40‐week WT and KI sural nerve cross sections showing myelinated fiber density (scalebar = 75 μm). (F) Quantification of 16‐week WT and KI sural nerve myelinated fiber densities (*n* = 4/genotype). (G) Cumulative frequency of axons by diameter in 16‐week WT and KI sural nerves. The inset shows comparison of all measured axon diameters between 16‐week WT and KI sural nerves (n = 4/genotype). (H) Representative immunofluorescent images of intraepidermal nociceptors compared between 16‐ and 40‐week WT and KI hindpaws (scalebar = 50 μm, inset scalebar = 10 μm). (I) Quantification of 16‐ and 40‐week WT and KI intraepidermal fiber densities (n = 5–6/genotype/timepoint). (J) Representative immunofluorescent images of intraepidermal nociceptors compared between 16 week WT and KO hindpaws (scalebar = 50 μm, inset scalebar = 10 μm). (K) Quantification of 16‐week WT and KO intraepidermal fiber densities (*n* = 4–5/genotype). All data are represented as mean ± SEM. (F, G, K) Analyzed via unpaired Student's *t*‐tests; (I) analyzed via two‐way ANOVA with Bonferroni comparisons between genotypes (*p < 0.05, ****p < 0.0001) and timepoints. [Color figure can be viewed at www.annalsofneurology.org]

Since SNAPs directly reflect the quantity of functional large, myelinated sensory axons within the recorded nerve, we examined ultrastructural changes in sections from the predominantly sensory modality sural nerve in KI mice at 16 weeks, corresponding to early SNAP and mechanosensation deficits (Figs 1B and 2C). Upon counting myelinated axons from ultrathin cross‐sections, we found that KI mice exhibit pronounced reductions in myelinated fiber densities relative to WT littermates (Fig 3E,F), indicating prominent sensory axon loss at this stage. Further analysis revealed preferential loss of large‐diameter axons among these fibers (Fig 3G), implicating compromised touch, vibration, and proprioception axons that likely contribute to KI mechanosensory and SNAP impairments.

Unmyelinated C‐fibers that innervate the epidermis are critical for nociceptive and thermal sensation. Injury to these intraepidermal fibers can lead to small fiber neuropathy, such as in the case of diabetic neuropathy, CMT1A, and fibromyalgia.^12^ We quantified intraepidermal fiber densities in KI plantar hindpaws relative to controls using PGP9.5 immunofluorescence at 16 and 40 weeks, timepoints corresponding to pre‐ and post‐onset of KI mouse thermal numbness (Fig 2D). KI, but not KO mice, exhibited consistent reductions in intraepidermal fiber density (Fig 3H–K), confirming small fiber neuropathy is a core feature of SCA3 KI peripheral neuropathology driven by mutant ATXN3 toxic gain‐of‐function. Collectively, these data demonstrate SCA3 KI mice develop peripheral ATXN3 accumulations and loss of both myelinated and unmyelinated sensory fibers, mirroring patient neuropathy pathology.^15^, ^16^, ^17^, ^18^

### SCA3 KI DRG Display Significant Sensory Neuron RNA Splicing Dysregulation without Overt Differential Gene Expression Changes

Loss of DRG sensory neurons has been reported in SCA3 patient peripheral pathology.^17^, ^24^, ^36^ Notwithstanding, transcriptional analysis relevant to SCA3 neuropathy phenotypes in DRG has never been performed, obscuring disease mechanisms. We conducted bulk RNA sequencing on 40‐week KI and WT DRG (Table S1, GSE320015). Surprisingly, Principal component analysis (PCA) based on differential gene expression showed no distinction between KI and WT DRG (Fig 4A). Differential gene expression analysis with a significance threshold of Padj <0.05 and log2FC > |1.5| also uncovered only 1 upregulated and 19 downregulated genes (Fig 4B).

**FIGURE 4:**
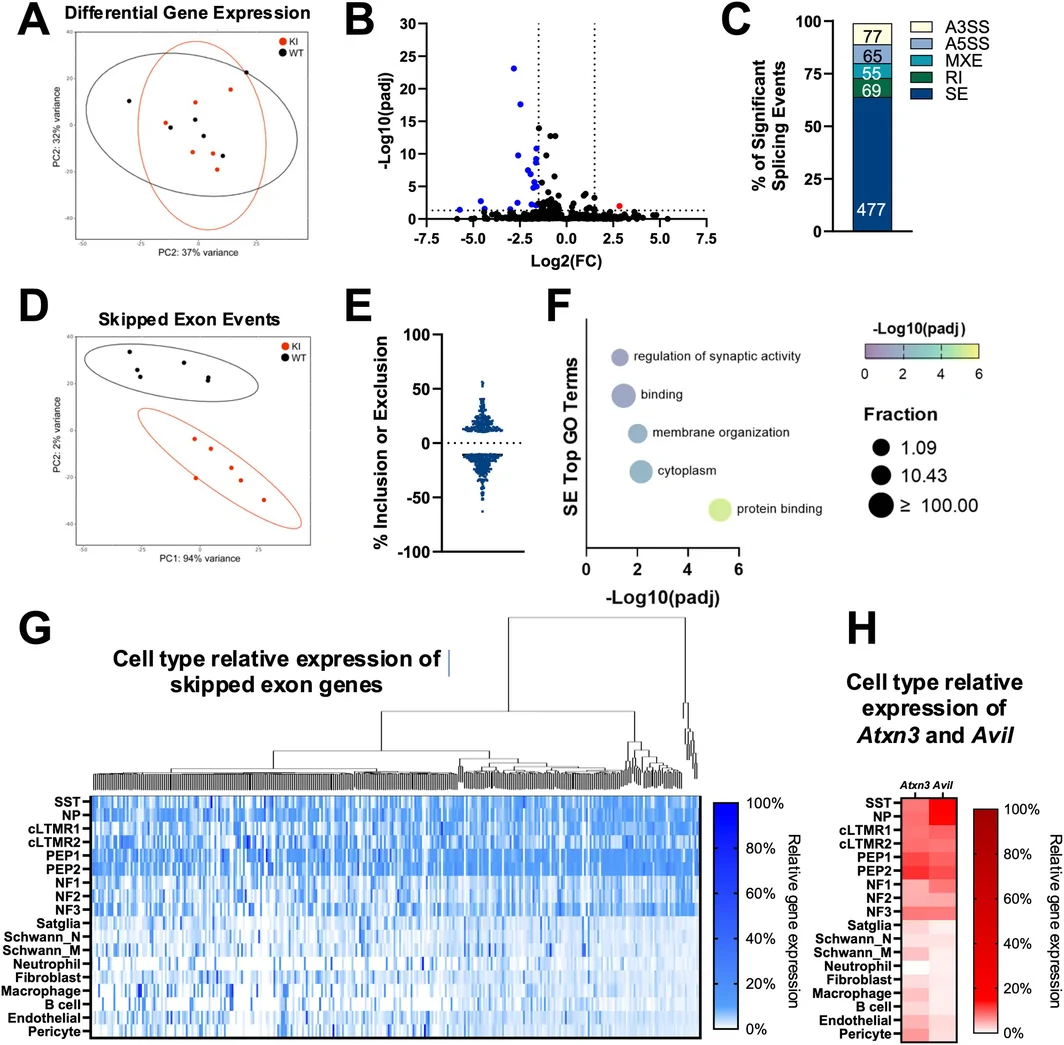
SCA3 KI DRG display significant sensory neuron RNA splicing dysregulation without overt differential gene expression changes. (A) PCA plot of gene expression data from 40‐week KI and WT DRG, with ellipses representing 95% confidence intervals. (B) Volcano plot highlighting upregulated (red) and downregulated (blue) differentially expressed genes in 40‐week KI DRG. (C) Percentage of significantly mis‐spliced skipped exon (SE), retained intron (RI), mutually exclusive exons (MXE), alternative 5′ splice site (A5SS), and alternative 3′ splice site (A3SS) events as a proportion of all significant splicing events in 40‐week KI and WT DRG datasets, FDR <0.05, ΔPSI >10%. (D) PCA plot of alternative RNA splicing data from 40‐week KI and WT DRG, with ellipses representing 95% confidence intervals. (E) Percentage of exon inclusion (positive) or exclusion (negative) for significant SE events. (F) Statistically significant GO terms derived from 40‐week KI DRG SE events. Fraction defines the fraction of GO term SE genes included in the dataset. (G) Heatmap of cell enrichment of 40‐week KI DRG SE events from cell type expression based on single‐nucleus RNA sequencing of mouse DRG.^44^ (H) Heatmap of Cell‐type relative gene expression of *Atxn3* and *Avil* corresponding to cell types defined in (G). SST = SSt + pruriceptors, NP = Mrgprd+ non‐peptidergic nociceptors, cLTMR1 = Fam19a4+/Th + C‐fiber LTMRs, cLTMR2 = Fam19a4+/Th‐ C‐LTMRs, PEP1 = Tac1+/Gpx3+ peptidergic nociceptors, PEP2 = Tac1+/Hpca+ peptidergic nociceptors. NF1 and NF2 = Pvalb+ proprioceptors, NF3 = Cadps2+ A𝛿‐ LTMRs, Satglia = satellite glia, Schwann_N = nonmyelinating Schwann cells, Schwann_M = myelinating Schwann cells. [Color figure can be viewed at www.annalsofneurology.org]

Given the limited mechanistic insights provided by differential gene expression analysis, we pivoted to assessing RNA splicing in our dataset. Studies have identified splicing dysregulation in pathogenic nervous system pathways across multiple SCAs, highlighting RNA splicing as a potential disease modulator in ataxias.^38^, ^39^ We evaluated 5 splicing types with FDR <0.05 and |PSI| > 0.1; 692 significant events were identified in KI DRG, with over half being skipped exons (Fig 4C). PCA of SE events showed clear segregation of KI from WT, highlighting splicing dysregulation's biological impact (Fig 4D). To characterize SE biology in KI DRG, we evaluated distribution bias between exon inclusion and exclusion within total SE events but found no differences (Fig 4E). Gene ontology (GO) analysis of KI DRG SE events highlighted terms regarding protein binding, membrane organization, and, importantly, regulation of synaptic activity (Fig 4F). As aberrant sensory neuron synaptic neurotransmission is a known contributor to peripheral neuropathy, we explored mis‐spliced genes constituting these synapse‐related GO terms. *Sybu* (mean PSI: WT = 29.62, KI = 61.03; FDR = 0.038) was significantly mis‐spliced, implicating disrupted axonal transport.^40^ We additionally identified aberrant splicing of *Ank2* (mean PSI: WT = 100, KI PSI = 83.28; *p* = 8.53^−06^) and *Agrn* (mean PSI: WT = 15.8, KI = 26.85; FDR = 0.022), key regulators of synaptic architecture, the latter of which has been implicated in rodent models of neuropathic pain.^41^, ^42^ Notably, *Nrg1* was also mis‐spliced (mean PSI: WT = 53.53, KI = 92.56; FDR = 0.014), a gene whose loss of function compromises axonal maintenance and sensory nerve conduction.^43^

Next, we sought further evidence that sensory neurons are the primary cell population exhibiting dysregulation in the KI DRG. Given the cellular heterogeneity of the DRG, comprising molecularly and functionally distinct sensory neuron subtypes alongside glia and immune cells, we mapped genes with significant SE events onto a published mouse DRG single‐nucleus RNA‐seq atlas.^44^ This analysis revealed enrichment across multiple sensory neuron subtypes, indicating that splice dysregulation is preferentially concentrated within neuronal populations relative to glia and immune cells (Fig 4G). Notably, these sensory neuron populations express *Atxn3* more highly than other non‐sensory DRG cells, connecting their propensity for splice dysregulation to the effects of mutant ATXN3 (Fig 4H). *Avil*, a gene encoding the ADVILLIN actin binding protein, is also highly expressed across DRG sensory neuron subtypes, marking it as an ideal promoter to selectively genetically target sensory neurons and assess their cell‐autonomous contributions to SCA3 peripheral neuropathy (Fig 4H).^45^

### *Selective Silencing of Mutant* Atxn3 *in Sensory Neurons Attenuates Peripheral Neuropathy Phenotypes and DRG Splicing Dysregulation*

To test the necessity of mutant ATXN3 in SCA3 sensory neuron pathology, we generated novel conditional KI *Atxn3* ^*cQ300/Q6*^ (cKI) mice with loxP sites flanking mutant *Atxn3* ^*Q300*^ exon 4 (Fig 5A). In the presence of Cre, exon 4 is excised, leading to multiple premature stop codons beginning within exon 5 residue 78, while sparing the normal *Atxn3* ^*Q6*^ allele (Fig S2). cKI mice were crossed with *Avil*‐*Cre* mice to achieve selective mutant ATXN3 silencing in sensory neurons.^45^ TdTomato reporter crossings confirmed specificity to DRG neurons (Figs 5B and S3A–C), and immunohistochemistry/protein quantification validated reduction of mutant ATXN3 (Fig 5C,D).

**FIGURE 5:**
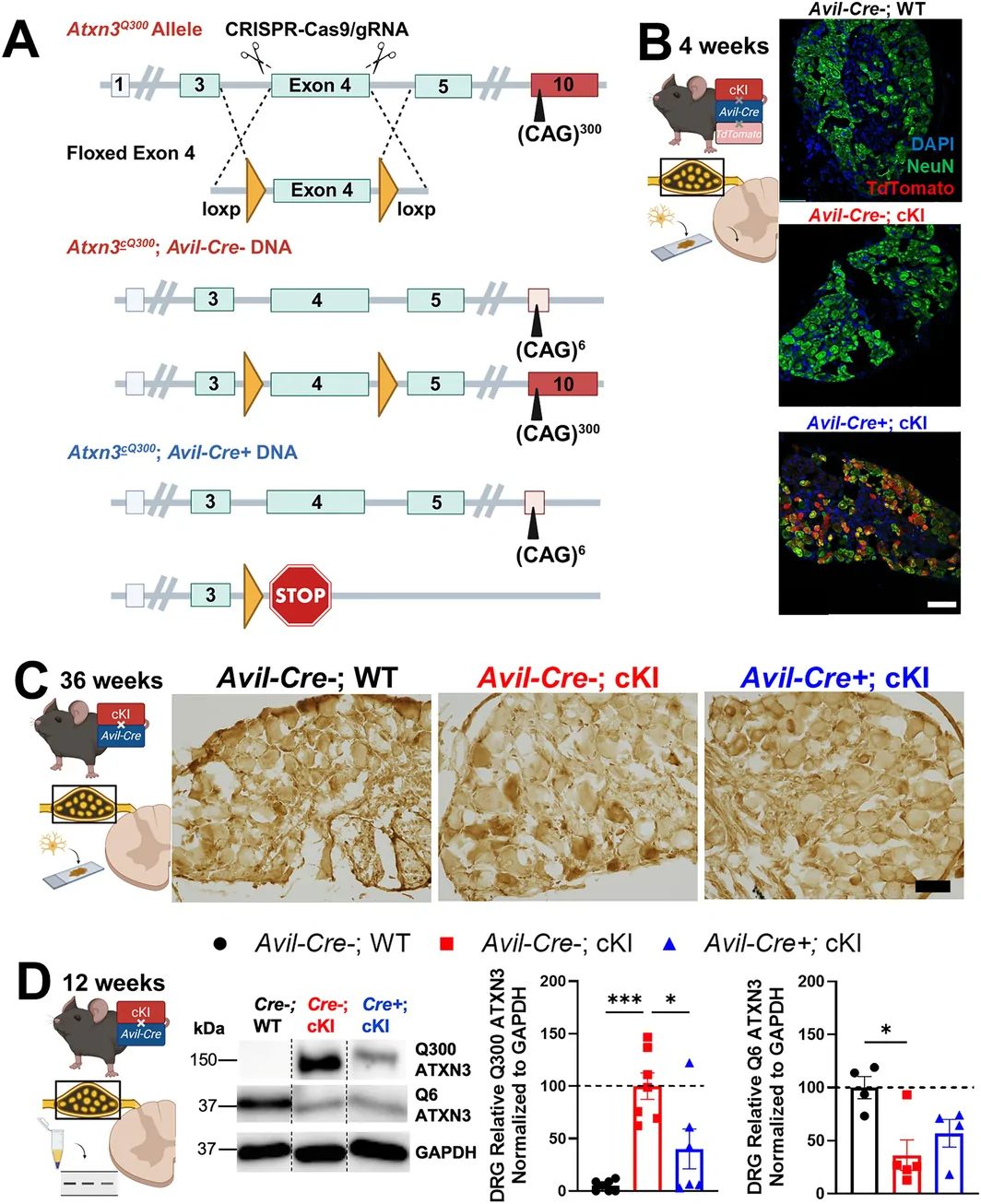
Sensory neuron mutant ATXN3 is selectively silenced in the novel *Avil‐Cre+;*cKI mouse. (A) Schematic of cKI mouse generation and floxed *Atxn3* ^*cQ300*^ allele silencing following Cre recombination. (B) Representative Immunofluorescent images showing DAPI, NeuN, and TdTomato signal in 4‐week *Avil‐Cre‐*;*TdTomato+;*WT, *Avil‐Cre‐;TdTomato+;*cKI, and *Avil‐Cre+;TdTomato+;*cKI lumbar DRG sections. (C) Representative images of anti‐ATXN3 immunohistochemistry from 36‐week *Avil‐Cre‐*;WT, *Avil‐Cre‐;*cKI, and *Avil‐Cre+*;cKI lumbar DRG (scalebar = 100 μm). (D) Representative images and quantification of 12‐week *Avil‐Cre‐*;WT, *Avil‐Cre‐;*cKI, and *Avil‐Cre+*;cKI lumbar DRG mutant expanded ATXN3 (1C2 antibody), wildtype ATXN3 (1H9 antibody), and GAPDH Western blot bands relative to controls. All data are represented as mean ± SEM. Western blot signals in (D) were quantified via one‐way ANOVA with Bonferroni comparisons between genotypes (*p < 0.05, ***p < 0.001). [Color figure can be viewed at www.annalsofneurology.org]

Longitudinal nerve conduction, coordination, and sensory assays were repeated in *Avil‐Cre‐;*WT, *Avil‐Cre‐;*cKI, and *Avil‐Cre+*;cKI mice at 12, 24, and 36 weeks. Remarkably, *Avil*‐*Cre*+;cKI mice showed rescued SNAPs/SNCVs, partial rescue of MNCV, and no improvement in CMAPs (Fig 6A–D). While ataxia symptoms such as a gait disturbances and motor incoordination can be partially caused by damage to somatosensory pathways, the role of sensory neuron repeat expansion pathology has never been directly assessed in relation to these motor disease features which are known to exacerbate fall risk and health deterioration. Astonishingly, balance beam and rotarod performance were improved or fully rescued in *Avil*‐*Cre*+;cKI mice (Fig 6E,F). Some open field locomotion deficits persisted, but tactile and thermal sensation were ameliorated (Figs 6G,H and S4A–C). Male, but not female, body weights were fully rescued (Fig S4D). These results suggest sensory neuropathy substantially contributes to sensorimotor deficits and highlight sensory neurons as targets for SCA3 therapy.

**FIGURE 6:**
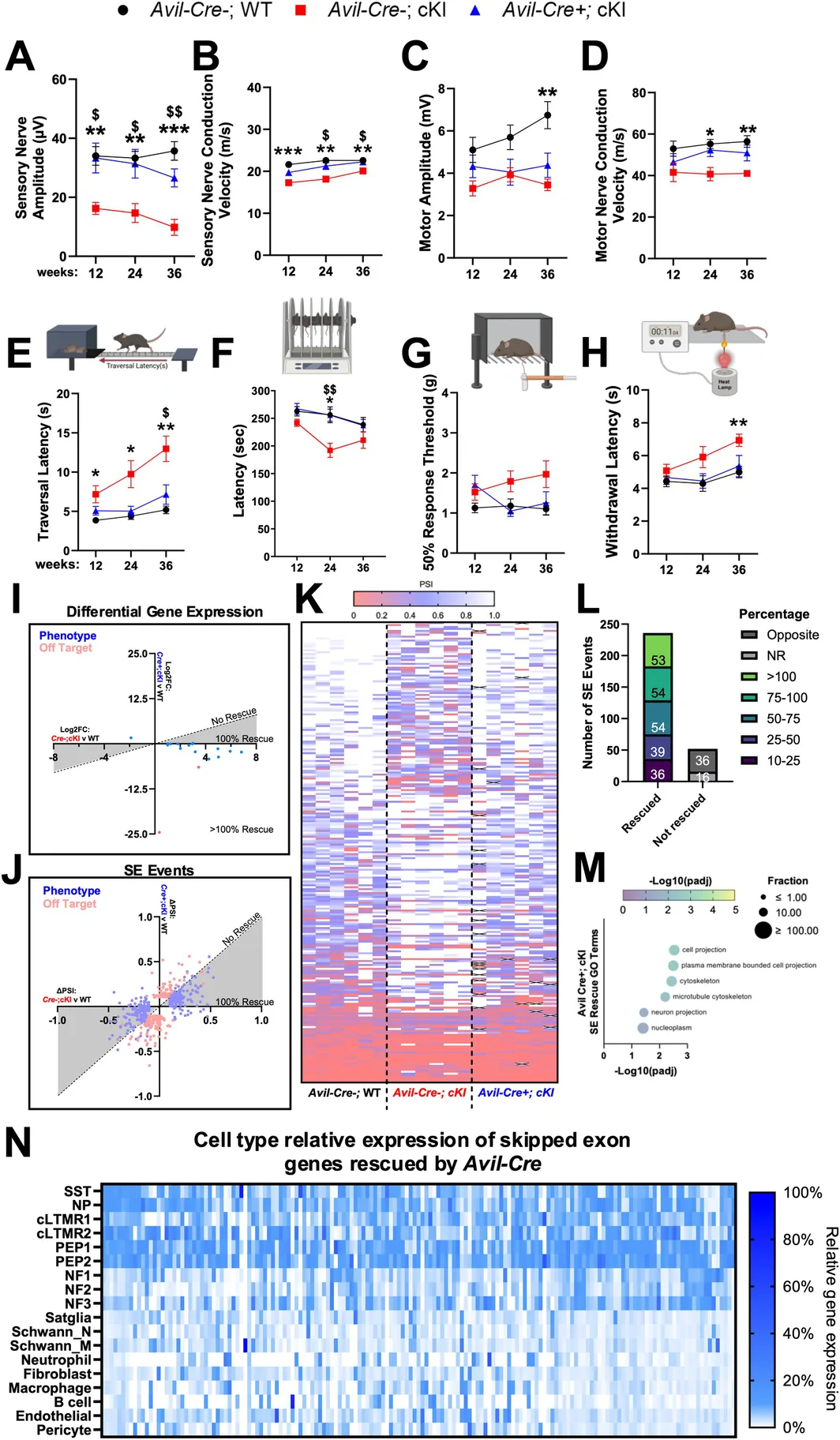
Selective silencing of mutant ATXN3 in sensory neurons ameliorates sensorimotor deficits and DRG RNA splicing dysregulation in cKI mice. (A) Sensory nerve amplitudes recorded from sural nerves compared between *Avil‐Cre‐*;WT, *Avil‐Cre‐;*cKI, and *Avil‐Cre+*;cKI mice at 12, 24, and 36 weeks (*n* = 6–10/genotype/timepoint). (B) Sensory nerve conduction velocities recorded from sural nerves compared between *Avil‐Cre‐*;WT, *Avil‐Cre‐;*cKI, and *Avil‐Cre+*;cKI mice at 12, 24, and 36 weeks (*n* = 6–10/genotype/timepoint). (C) Compound muscle action potential amplitudes recorded from sciatic nerves compared between *Avil‐Cre‐*;WT, *Avil‐Cre‐;*cKI, and *Avil‐Cre+*;cKI mice at 12, 24, and 36 weeks (*n* = 6–9/genotype/timepoint). (D) Motor nerve conduction velocities recorded from sciatic nerves compared between *Avil‐Cre‐*;WT, *Avil‐Cre‐;*cKI, and *Avil‐Cre+*;cKI mice at 12, 24, and 36 weeks (*n* = 6–9/genotype/timepoint). (E) Latency to traverse balance beam compared between *Avil‐Cre‐*;WT, *Avil‐Cre‐;*cKI, and *Avil‐Cre+*;cKI mice at 12, 24, and 36 weeks (*n* = 8–12/genotype/timepoint). (F) Latency to fall off accelerating rotarod compared between *Avil‐Cre‐*;WT, *Avil‐Cre‐;*cKI, and *Avil‐Cre+*;cKI mice at 12, 24, and 36 weeks (*n* = 8–11/genotype/timepoint). (G) Von Frey 50% response thresholds compared between *Avil‐Cre‐*;WT, *Avil‐Cre‐;*cKI, and *Avil‐Cre+*;cKI mice at 12, 24, and 36 weeks (*n* = 6–12/genotype/timepoint). (H) Thermal hindpaw withdrawal latencies compared between *Avil‐Cre‐*;WT, *Avil‐Cre‐;*cKI, and *Avil‐Cre+*;cKI mice at 12, 24, and 36 weeks (*n* = 8–12/genotype/timepoint). All data are represented as mean ± SEM. All data were analyzed via two‐way ANOVA with Bonferroni comparisons between *Avil‐Cre‐;*WT and *Avil‐Cre‐;*cKI (*p < 0.05, **p < 0.01, ***p < 0.001), *Avil‐Cre‐;*cKI and *Avil‐Cre+;*cKI mice ($*p* < 0.05, $$*p* < 0.01), and *Avil‐Cre‐*; WT and *Avil‐Cre+*; cKI mice. (I) Plot comparing Log2FC between *Avil‐Cre‐*;cKI and *Avil‐Cre‐*;WT mice (x‐axis) versus Log2FC between *Avil‐Cre* +;cKI and *Avil‐Cre*‐;WT mice (y‐axis) for all differentially expressed genes (log2FC > |1.5|, Padj <0.05) from each comparison (*Avil‐Cre* ‐;cKI vs *Avil‐Cre*‐;WT = Phenotype *Avil‐Cre*+;cKI vs *Avil‐Cre* ‐;WT = Off Target). (J) Plot comparing ΔPSI between *Avil‐Cre*‐;cKI and *Avil‐Cre*‐;WT mice (x‐axis) versus ΔPSI between *Avil‐Cre* +;cKI and *Avil‐Cre* ‐;WT mice (y‐axis) for all significant SE events (ΔPSI>10%, FDR <0.05) from each comparison (*Avil‐Cre* ‐;cKI vs *Avil‐Cre*‐;WT = Phenotype, *Avil‐Cre*+;cKI vs *Avil‐Cre*‐;WT = Off Target). Events that cluster around the x‐axis show 100% rescue while events that cluster along the dotted line show no rescue. (K) Heatmap showing PSI value of SE events significantly dysregulated in *Avil‐Cre*‐;cKI mice and rescued in *Avil‐Cre*+;cKI mice for every DRG sample. Crossed out boxes indicate events that were not detected in a given sample. (L) Bar plot showing number of SE events rescued by *Avil‐Cre*. Rescued events categorized based on the percentage ΔPSI rescue. NR = not rescued. (M) Top GO terms derived from SE events rescued by *Avil‐Cre*. Fraction is the proportion of rescued SE event genes in a given GO term. (N) Heatmap showing cell enrichment of SE events rescued in *Avil‐Cre*+;cKI DRG relative to *Avil‐Cre*‐;cKI based on cell type expression data from single nucleus RNA sequencing of mouse DRG.^44^ Nerve conduction and behavioral data are represented as mean ± SEM. And analyzed via two‐way ANOVA with Bonferroni comparisons between *Avil‐Cre*‐; WT and *Avil‐Cre*‐;cKI (*p < 0.05, **p < 0.01, ***p < 0.001), and *Avil‐Cre*‐;cKI and *Avil‐Cre*+;cKI mice ($*p* < 0.05, $$*p* < 0.01). SST = SSt + pruriceptors, NP = Mrgprd+ non‐peptidergic nociceptors, cLTMR1 = Fam19a4+/Th + C‐fiber LTMRs, cLTMR2 = Fam19a4+/Th‐ C‐LTMRs, PEP1 = Tac1+/Gpx3+ peptidergic nociceptors, PEP2 = Tac1+/Hpca+ peptidergic nociceptors. NF1 and NF2 = Pvalb+ proprioceptors, NF3 = Cadps2+ A𝛿‐ LTMRs, Satglia = satellite glia, Schwann_N = nonmyelinating Schwann cells, Schwann_M = myelinating Schwann cells. [Color figure can be viewed at www.annalsofneurology.org]

Considering the splicing dysregulation uncovered in KI DRG (Fig 4), we investigated the association between DRG alternative RNA splicing and the functional restoration achieved by sensory neuron mutant ATXN3 silencing in *Avil‐Cre+*;cKI mice. We conducted bulk RNA sequencing on DRG from *Avil‐Cre‐*;WT, *Avil‐Cre‐*;cKI, and *Avil‐Cre+*;cKI at 36 weeks, a timepoint with robust rescue of multiple sensory neuropathy readouts (Table S2, GSE329323). Consistent with our 40‐week KI dataset, differential gene expression analysis with a significance threshold of Log2FC > |1.5| and Padj <0.05 yielded only 16 differentially expressed genes between *Avil‐Cre‐*;WT and *Avil‐Cre‐*;cKI DRG, with 6 showing full rescue in *Avil‐Cre+*;cKI (Fig 6I). Alternative splicing analysis showed significant splicing events across all genotype comparisons, with the majority being SE events without exon inclusion or exclusion bias (Fig S4E,F). SE event analysis revealed 258 significant SE events (ΔPSI>10%, FDR <0.05) between *Avil‐Cre‐*;cKI and *Avil‐Cre‐*;WT DRG, 206 of which were fully rescued in *Avil‐Cre+*;cKI (Fig 6J–L) (rescue defined as an average PSI in the *Avil‐Cre+*;cKI mice >10% different from that in *Avil‐Cre‐*;cKI mice in the direction of *Avil‐Cre‐*;WT mice). GO analysis of rescued SE events produced neuron projection‐related terms, suggesting a sensory neuron bias in splicing rescue (Fig 6M). We mapped rescued SE genes onto a published mouse DRG single‐nucleus RNA‐seq atlas^44^ and confirmed that these genes were enriched in DRG sensory neuron populations (Fig 6N). These findings highlight splicing dysregulation as a potential key modulator of sensory neuron‐ driven neuropathic phenotypes in SCA3 and support the efficacy of mutant ATXN3 silencing in sensory neurons in ameliorating these alterations.

## Discussion

This study establishes a direct mechanistic link between polyglutamine expanded ATXN3 and peripheral neuropathy in SCA3, leveraging genetically modified mouse models to overcome clinical research limitations in a rare repeat expansion disorder. Our SCA3 KI model robustly recapitulates peripheral disease features phenocopying those observed in patients, including reduced nerve amplitudes, deficits in balance and sensation, ATXN3 accumulation in peripheral nerves, and loss of sensory fibers.^3^, ^13^, ^15^, ^16^, ^17^, ^18^, ^20^, ^24^, ^36^ These neuropathic phenotypes were accompanied by sensory neuron‐enriched splicing abnormalities in KI DRG, representing, to our knowledge, the first evidence of such RNA dysregulation in DRG within a repeat expansion disorder. These impairments are all driven by mutant ATXN3 toxic gain‐of‐function, as KO mice do not exhibit similar deficits. Building on these findings, silencing mutant ATXN3 in sensory neurons rescued clinically relevant peripheral phenotypes in cKI mice, including SNAP reductions, impairments in balance and coordination, sensation/reflex deficits, and DRG RNA splicing. These findings highlight sensory neurons as critical mediators of SCA3 peripheral dysfunction and establish their targeting as a promising therapeutic strategy.

Advances in animal modeling have greatly accelerated therapeutic development for diverse neurodegenerative diseases, yet modeling peripheral neuropathy in repeat expansion disorders have lagged behind due to limited model characterization. Our rigorous peripheral neuropathy phenotyping in the SCA3 KI mouse provides a translational foundation for therapeutic testing, paralleling model advances in ATTR, GAN, and CMT1A, which enabled preclinical and clinical trials for those diseases. Notably, GAN gene replacement strategies and antisense oligonucleotides (ASOs) targeting *PMP22* and *TTR* in CMT1A and ATTR, respectively, all improve peripheral neuropathy impairments in rodent models.^9^, ^46^, ^47^ Preclinical work focusing on CNS pathology in SCA3 mouse models has demonstrated that gene silencing of ATXN3 alleviates various progressive brain pathologies and motor impairments.^13^, ^48^ Similarly, ASO‐mediated suppression of ATXN1 rescues alternative splicing dysregulation in disease‐relevant CNS pathways in an SCA1 model.^38^ However, therapeutic efficacy of anti‐*ATXN3* ASOs have never been preclinically assessed for treatment of SCA3 peripheral neuropathy due to the lack of peripheral mouse model characterizations. With our findings of peripheral disease in SCA3 KI mice, future studies can directly test the impact of intrathecally‐administered anti‐ATXN3 ASOs. Alternatively, adeno‐associated virus (AAV)‐delivered microRNAs targeting *ATXN3* could be tested to achieve durable, long‐term suppression with a single administration.^48^ While sensory neuropathy‐targeted therapies would likely relieve disability in SCA3, it must be noted that treatment combination strategies may be necessary to address CNS pathology‐driven clinical features in complement to sensory neuron‐related disease features. Our approach also prompts evaluation of peripheral phenotypes in models of other repeat expansion diseases, such as SCA1, SCA2, SCA6, SCA7, SCA27B, CANVAS, Friedreich's Ataxia, and SBMA, where sensory neuropathy is prevalent but preclinical and clinical assessments remain rare.^3^, ^4^, ^6^, ^16^, ^24^, ^49^ The use of gold standard, clinically relevant nerve conduction studies and behavioral assays in preclinical studies will strengthen the translational significance and possibly guide future therapeutic trials.^17^, ^33^

Commonly used clinical trial biomarker endpoints in SCA3, including the Scale for the Assessment and Rating of Ataxia, MRI volumetry, and serum neurofilament light, are challenged by requirements for large sample sizes and limited in their ability to capture peripheral involvement, ultimately hindering effective monitoring of disease progression in light of our studies.^50^ These challenges underscore a need for more sensitive biomarkers. Our findings have direct biomarker implications, as robust reductions in SNAPs are seen across multiple SCAs and represent early and progressive peripheral phenotypes in SCA3 that may even precede ataxia onset.^3^, ^17^, ^31^ The utility of SNAPs as sensitive, treatment‐responsive biomarkers is underscored in GAN, where gene replacement therapy has been shown to recover SNAPs from unrecordable in severely ill patients to clearly measurable amplitudes upon treatment.^9^ While additional patient studies are needed to establish longitudinal associations between sensorimotor nerve conduction deterioration and standard SCA3 clinical metrics, tracking these measures could enable more precise monitoring of disease progression and therapeutic response than broader subjective clinical scales.

There are several important limitations to our studies that will need to be addressed in future preclinical programs. First, our KI mouse model expresses the murine rather than human *ATXN3* gene, which may limit the direct translatability of our findings to human SCA3. To address this, future studies assessing peripheral neuropathy in SCA3 YACQ84 mice, which express the full‐length human mutant *ATXN3* gene under the human *ATXN3* promoter and recapitulate SCA3 brain pathologies and motor impairments, will be critical in clarifying the relevance of our work to human therapeutic strategies.^14^, ^26^ Second, although our genetic approach achieves robust silencing of mutant ATXN3 in the majority of sensory neurons at the study endpoint, this study does not determine the threshold of ATXN3 knockdown required to achieve meaningful behavioral rescue. Additional experiments using gene silencing therapeutics will be necessary to establish the minimal level of mutant ATXN3 reduction in sensory neurons needed to confer therapeutic benefit. Finally, future studies employing inducible, cell type specific approaches are needed to rigorously define the critical windows for effective therapeutic intervention in SCA3 models.

## Author Contributions

J.P.M. and H.S.M contributed to the conception and design of the study; J.P.M., J.M.H., J.E., M.S., T.A., A.N., and A.F.P. contributed to the acquisition and analysis of data; J.P.M., J.E., and H.S.M. contributed to drafting the text or preparing the figures.

## Potential Conflicts of Interest

Nothing to report.

## Supporting information


**Figure S1** Mutant ATXN3 toxic *gain*‐*of* function causes locomotion and weight reductions without anxiety or joint position challenges. (A) Open field total locomotion, rears, and % time spent in the center of the open field assessed in 16‐, 24‐, and 40‐week KI and 8‐, 16‐, and 24‐week KO mice compared to WT controls (KI: *n* = 23–24/genotype/timepoint; KO: *n* = 8–12/genotype/timepoint). (B) Open field rears assessed in 16‐, 24‐, and 40‐week KI and 8‐, 16‐, and 24‐week KO mice compared to WT controls (KI: *n* = 23–24/genotype/timepoint; KO: *n* = 8–12/genotype/timepoint). (C) Percentage of time spent in the center of the open field assessed in 16‐, 24‐, and 40‐week KI and 8‐, 16‐, and 24‐week KO mice compared to WT controls (KI: n = 23–24/genotype/timepoint; KO: *n* = 8–12/genotype/timepoint). (D) Hindlimb clasping scores obtained in 40‐week KI and 24‐week KO mice compared to WT controls (KI: *n* = 6/genotype; KO: n = 4–6/genotype). (E) Male and female body weight measured in 16‐, 24‐, and 40‐week KI and 8‐, 16‐, and 24‐week KO compared to WT controls (KI: *n* = 7–15/genotype/timepoint/sex; KO: *n* = 2–10/genotype/timepoint/sex). All data are represented as mean ± SEM. (A‐C) and (E) were analyzed via two‐way ANOVA with Bonferroni comparisons between genotypes (*p < 0.05, **p < 0.01, ***p < 0.001) and timepoints # *p* < 0.05), while (D) was analyzed with Student's unpaired t‐test.
**Figure S2**. Generation of SCA3 conditional “OFF” knock‐in mouse model. (A) SCA3 conditional Knock‐In Q300 mice (cKI), have loxP sites flanking exon 4 upstream of the hyper‐expanded 300 CAG repeats in exon 10 of murine *Atxn3*. The *Atxn3* Q6 allele remains unaltered, with an endogenous 6 CAG repeats in exon 10. The location of loxP sites was verified via long‐read sequencing (inset). “F” and “R” arrows indicate position of primers used for PCR validation of exon 4 recombination. (B) In cells without Cre, exon 4 remains intact. In cells with Cre, deletion of exon 4 on the expanded allele causes a frameshift resulting in three stop codons. (C) PCR amplification of exon 4 shows a WT allele (●) in all 36‐week DRG genomic DNA samples. *Avil‐Cre*‐;cKI and *Avil‐Cre*+;cKI samples have a slightly larger allele signifying loxP insertion (■). Only *Avil‐Cre+*;cKI mice show a reduction in the intensity of the top band and the appearance of a smaller band (♦) representing the removal of exon 4.
**Figure S3**. *Avil‐Cre; TdTomato* reporter mouse demonstrates robust Cre expression in sensory neurons/axons and limited Cre expression varied across brain structures. (A) Representative Immunofluorescent images showing DAPI, NeuN, and TdTomato in 4‐week *Avil‐Cre+;TdTomato +* DRG cross sections (scalebar = 50 μm). (B) Representative Immunofluorescent images showing DAPI, S100β, and TdTomato in 4‐week *Avil‐Cre+;TdTomato +* sciatic nerve cross sections (scalebar = 75 μm). (C) Representative Immunofluorescent images showing DAPI, CGRP, and TdTomato in 4‐week *Avil‐Cre+;TdTomato +* lumbar spinal cord cross sections (scalebar = 100 μm). (D) Immunofluorescent images of 4‐week *Avil‐Cre+;TdTomato +* brain sections illustrating variability in TdTomato reporter expression across biological replicates. Insets show the whole cerebellum (CB, solid line), magnifications of a cerebellar lobule, and the somatosensory cortex (SM CTX, dashed line) and cerebellum (CB) TdTomato expression (scalebar = 1 mm; whole cerebellum inset scalebar = 1 mm; cerebellar lobule inset scale bar = 100 μm; somatosensory cortex inset scalebar = 250 μm). a, a’, and b delineate corresponding brain inset panels.
**Figure S4**. Silencing mutant ATXN3 in sensory neurons does not improve locomotion but attenuates male weight reduction and alters alternative splicing in DRG. (A) Open field total locomotion compared between *Avil‐Cre‐*;WT, *Avil‐Cre‐*;cKI, and *Avil‐Cre+*;cKI mice at 12, 24, and 36 weeks (*n* = 8–11/genotype/timepoint). (B) Open field rears compared between *Avil‐Cre‐*;WT, *Avil‐Cre‐*;cKI, and *Avil‐Cre+*;cKI mice at 12, 24, and 36 weeks (*n* = 8–11/genotype/timepoint). (C) Percentage of time spent in the center of the open field compared between *Avil‐Cre‐*;WT, *Avil‐Cre‐*;cKI, and *Avil‐Cre+*;cKI mice at 12, 24, and 36 weeks (*n* = 8–11/genotype/timepoint). (D) Male and female body weight compared between *Avil‐Cre‐*;WT, *Avil‐Cre‐*;cKI, and *Avil‐Cre+*;cKI mice at 12, 24, and 36 weeks (*n* = 2–7/genotype/timepoint). (E) Proportional amount of significant splicing events (ΔPSI>10%, FDR <0.05) from each comparison among *Avil‐Cre‐*; WT, *Avil‐Cre‐*; cKI, and *Avil‐Cre+*; cKI DRG. Numbers represent significant event count for each event type: Skipped exon (SE), retained introns (RI), mutually exclusive exons (MXE), alternative 5′ splice site (A5SS), and alternative 3′ splice site (A3SS). (F) Percent Inclusion or exclusion of all significant skipped exon events from each comparison among *Avil‐Cre*‐; WT, *Avil‐Cre‐*; cKI, and *Avil‐Cre+*; cKI DRG. All data are represented as mean ± SEM and were analyzed via two‐way ANOVA with Bonferroni comparisons between *Avil‐Cre‐*; WT and *Avil‐Cre‐*; cKI (*p < 0.05, **p < 0.01), *Avil‐Cre‐*; cKI and *Avil‐Cre+*; cKI ($*p* < 0.05, $$$*p* < 0.001), and *Avil‐Cre‐*; WT and *Avil‐Cre+*; cKI (#*p* < 0.05, ##*p* < 0.01) mice.
**Table S1**. 40‐week KI DRG RNA samples
**Table S2**. 36‐week Avil‐Cre; cKI DRG RNA samples

## Data Availability

All data associated with this study are present in the paper, Supporting Information, and Supporting Data Values. Raw data images files are available at DANDI:001621, https://doi.org/10.48324/dandi.001621/0.251208.0008. RNA sequencing data is available in following online repositories: GSE320015 and GSE329323.
