# Supplementary material for: Establishing Sensory Neurons as Therapeutic Targets in Peripheral Neuropathy Driven by Polyglutamine Expanded Murine ATXN3
Source: Ann Neurol. 2026 Jul 31;100(5):1039–54. doi: 10.1002/ana.78317 (PMC13688050; doi:10.1002/ana.78317)
Supplement: Supplementary file 1 [file ANA-100-1039-s001.docx]

**SUPPLEMENTARY MATERIAL**

**Methods**

**Open Field testing**

Open Field tests were conducted in a photobeam open field apparatus (San Diego Instruments, San Diego, CA) to measure free-roam locomotor and exploratory activity. Locomotion was evaluated by measuring the number of x/y-axis beam breaks during a 30-minute trial. In the same trial, rearing was assessed by measuring the number of z-axis beam breaks as previously described ^25^. The time that mice spent in the open field center was divided by the time spent in the periphery to assess anxiety.

**Clasping**

Mice were held upside down by the base of the tail and suspended immediately above the roof of their home cage for a period of at least 10 seconds across two trials. Mice were given approximately one minute of rest in between trials. Trials were recorded for later quantification, in which hindlimb clasping scores were assigned according to the following criteria: 0 - No hindlimb clasping; 1 – one hindlimb clasped for less than 50% of the time; 2 – clasping one hindlimb for at least 50% of the time; or 3 - clasping both hindlimbs at least 50% of the time.

**Figures**

**Fig. S1. Mutant ATXN3 toxic *gain*-*of* function causes locomotion and weight reductions without anxiety or joint position challenges. (A)** Open field total locomotion, rears, and % time spent in the center of the open field assessed in 16-, 24-, and 40-week KI and 8-, 16-, and 24-week KO mice compared to WT controls (KI: n = 23–24/genotype/timepoint; KO: n = 8–12/genotype/timepoint). **(B)** Open field rears assessed in 16-, 24-, and 40-week KI and 8-, 16-, and 24-week KO mice compared to WT controls (KI: n = 23–24/genotype/timepoint; KO: n = 8–12/genotype/timepoint). **(C)** Percentage of time spent in the center of the open field assessed in 16-, 24-, and 40-week KI and 8-, 16-, and 24-week KO mice compared to WT controls (KI: n = 23–24/genotype/timepoint; KO: n = 8–12/genotype/timepoint). **D)** Hindlimb clasping scores obtained in 40-week KI and 24-week KO mice compared to WT controls (KI: n = 6/genotype; KO: n = 4–6/genotype). **(E)** Male and female body weight measured in 16-, 24-, and 40-week KI and 8-, 16-, and 24-week KO compared to WT controls (KI: n = 7–15/genotype/timepoint/sex; KO: n = 2–10/genotype/timepoint/sex). All data are represented as mean ± SEM. (A-C) and (E) were analyzed via two-way ANOVA with Bonferroni comparisons between genotypes (* p < 0.05, ** p < 0.01, *** p < 0.001) and timepoints # p < 0.05), while (D) was analyzed with Student’s unpaired t-test.

**Fig. S2. Generation of SCA3 conditional “OFF” knock-in mouse model. (A)** SCA3 conditional Knock-In Q300 mice (cKI), have loxP sites flanking exon 4 upstream of the hyper-expanded 300 CAG repeats in exon 10 of murine *Atxn3*. The *Atxn3* Q6 allele remains unaltered, with an endogenous 6 CAG repeats in exon 10. The location of loxP sites was verified via long-read sequencing (inset). “F” and “R” arrows indicate position of primers used for PCR validation of exon 4 recombination. **(B)** In cells without Cre, exon 4 remains intact. In cells with Cre, deletion of exon 4 on the expanded allele causes a frameshift resulting in three stop codons.

**(C)** PCR amplification of exon 4 shows a WT allele (●) in all 36-week DRG genomic DNA samples. *Avil-Cre*-;cKI and *Avil-Cre*+;cKI samples have a slightly larger allele signifying loxP insertion (■). Only *Avil-Cre+*;cKI mice show a reduction in the intensity of the top band and the appearance of a smaller band (♦) representing the removal of exon 4.

**Fig. S3. *Avil-Cre; TdTomato* reporter mouse demonstrates robust Cre expression in sensory neurons/axons and limited Cre expression varied across brain structures. (A)** Representative Immunofluorescent images showing DAPI, NeuN, and TdTomato in 4-week *Avil-Cre+;TdTomato+* DRG cross sections (scalebar = 50 µm). **(B)** Representative Immunofluorescent images showing DAPI, S100β, and TdTomato in 4-week *Avil-Cre+;TdTomato+* sciatic nerve cross sections (scalebar = 75 µm). **(C)** Representative Immunofluorescent images showing DAPI, CGRP, and TdTomato in 4-week *Avil-Cre+;TdTomato+* lumbar spinal cord cross sections (scalebar = 100 µm). **(D)** Immunofluorescent images of 4-week *Avil-Cre+;TdTomato+* brain sections illustrating variability in TdTomato reporter expression across biological replicates. Insets show the whole cerebellum (CB, solid line), magnifications of a cerebellar lobule, and the somatosensory cortex (SM CTX, dashed line) and cerebellum (CB) TdTomato expression (scalebar = 1 mm; whole cerebellum inset scalebar = 1 mm; cerebellar lobule inset scale bar = 100 µm; somatosensory cortex inset scalebar = 250 µm). a, a’, and b delineate corresponding brain inset panels.

**Fig. S4** **. Silencing mutant ATXN3 in sensory neurons does not improve locomotion but attenuates male weight reduction and alters alternative splicing in DRG. (A)** Open field total locomotion compared between *Avil-Cre-*;WT, *Avil-Cre-*;cKI, and *Avil-Cre+*;cKI mice at 12, 24, and 36 weeks (n= 8–11/genotype/timepoint). **(B)** Open field rears compared between *Avil-Cre-*;WT, *Avil-Cre-*;cKI, and *Avil-Cre+*;cKI mice at 12, 24, and 36 weeks (n= 8–11/genotype/timepoint). **(C)** Percentage of time spent in the center of the open field compared between *Avil-Cre-*;WT, *Avil-Cre-*;cKI, and *Avil-Cre+*;cKI mice at 12, 24, and 36 weeks (n= 8–11/genotype/timepoint). **(D)** Male and female body weight compared between *Avil-Cre-*;WT, *Avil-Cre-*;cKI, and *Avil-Cre+*;cKI mice at 12, 24, and 36 weeks (n= 2–7/genotype/timepoint). **(E)** Proportional amount of significant splicing events (ΔPSI>10%, FDR<0.05) from each comparison among *Avil-Cre-*; WT, *Avil-Cre-*; cKI, and *Avil-Cre+*; cKI DRG. Numbers represent significant event count for each event type: Skipped exon (SE), retained introns (RI), mutually exclusive exons (MXE), alternative 5’ splice site (A5SS), and alternative 3’ splice site (A3SS).

**(F)** Percent Inclusion or exclusion of all significant skipped exon events from each comparison among *Avil-Cre*-; WT, *Avil-Cre-*; cKI, and *Avil-Cre+*; cKI DRG.

All data are represented as mean ± SEM and were analyzed via two-way ANOVA with Bonferroni comparisons between *Avil-Cre-*; WT and *Avil-Cre-*; cKI (*p<0.05, **p<0.01), *Avil-Cre-*; cKI and *Avil-Cre+*; cKI ($p<0.05, $$$p<0.001), and *Avil-Cre-*; WT and *Avil-Cre+*; cKI (#p<0.05, ##p<0.01) mice.

**Supplementary Tables**

**Supplemental Table 1. 40-week KI DRG RNA samples**

| **Mouse Number** | **Genotype** | **Sex** | **RIN of RNA sample** | **Library Name in GEO** | **Read Counts** |
| --- | --- | --- | --- | --- | --- |
| b535.0 | WT | M | 9.0 | 12261-JM-1 | 111107254 |
| b526.2 | WT | M | 8.8 | 12661-JM-2 | 106234835 |
| b526.4 | WT | M | 7.7 | 12661-JM-3 | 121137238 |
| b553.0 | WT | F | 8.1 | 12661-JM-4 | 93091987 |
| b533.1 | WT | F | 8.6 | 12661-JM-5 | 89825345 |
| b534.0 | WT | F | 8.7 | 12661-JM-6 | 99136880 |
| b485.4 | KI | M | 7.1 | 12661-JM-7 | 118632439 |
| b490.3 | KI | M | 7.8 | 12661-JM-8 | 95671112 |
| b553.2 | KI | F | 8.8 | 12661-JM-9 | 96639767 |
| b490.0 | KI | F | 8.3 | 12661-JM-10 | 94209248 |
| b490.2 | KI | F | 7.9 | 12661-JM-11 | 90374896 |
| b522.6 | KI | F | 8.7 | 12661-JM-12 | 97262798 |

**Supplemental Table 2. 36-week *Avil-Cre;* cKI DRG RNA samples**

| **Mouse Number** | **Genotype** | **Sex** | **RIN of RNA sample** | **Library Name in GEO** | **Read Counts** |
| --- | --- | --- | --- | --- | --- |
| b601.2 | *Avil-Cre-;* WT | M | 8.2 | 15436-JM-1 | 89386031 |
| b571.0 | *Avil-Cre-;* WT | F | 8.6 | 15436-JM-2 | 84765025 |
| b642.5 | *Avil-Cre-;* WT | F | 8.8 | 15436-JM-3 | 104405259 |
| b572.0 | *Avil-Cre-;* WT | M | 9.0 | 15436-JM-4 | 95060702 |
| b661.0 | *Avil-Cre-;* WT | M | 8.3 | 15436-JM-5 | 93537721 |
| b634.0 | *Avil-Cre-;* WT | F | 8.3 | 15436-JM-6 | 99019611 |
| b548.2 | *Avil-Cre-;* cKI | M | 8.2 | 15436-JM-7 | 73228084 |
| b642.1 | *Avil-Cre-;* cKI | M | 8.3 | 15436-JM-8 | 92566041 |
| b642.4 | *Avil-Cre-;* cKI | M | 8.6 | 15436-JM-9 | 86588980 |
| b571.4 | *Avil-Cre-;* cKI | M | 9.0 | 15436-JM-10 | 88411487 |
| b687.1 | *Avil-Cre-;* cKI | F | 8.1 | 15436-JM-11 | 88960309 |
| b732.0 | *Avil-Cre-;* cKI | M | 8.6 | 15436-JM-12 | 81166197 |
| b548.3 | *Avil-Cre+;* cKI | M | 7.4 | 15436-JM-13 | 86484139 |
| b566.3 | *Avil-Cre+;* cKI | M | 8.4 | 15436-JM-14 | 82279930 |
| b661.7 | *Avil-Cre+;* cKI | M | 8.8 | 15436-JM-15 | 84826928 |
| b732.2 | *Avil-Cre+;* cKI | F | 8.1 | 15436-JM-17 | 101116840 |
| b601.1 | *Avil-Cre+;* cKI | F | 8.7 | 15436-JM-18 | 86960991 |
| b572.1 | *Avil-Cre+;* cKI | F | 8.9 | 15436-JM-19 | 86155961 |
